# DNA barcoding of southern African crustaceans reveals a mix of invasive species and potential cryptic diversity

**DOI:** 10.1371/journal.pone.0222047

**Published:** 2019-09-16

**Authors:** Bezeng S. Bezeng, Herman F. van der Bank

**Affiliations:** 1 African Centre for DNA Barcoding, University of Johannesburg, Auckland Park, Johannesburg, South Africa; 2 School of Mathematics & Natural Sciences, University of Venda, P. Thohoyandou, Venda, South Africa; 3 Department of Geography, Environmental Management and Energy Studies, University of Johannesburg, Auckland Park, Johannesburg, South Africa; Nanjing Agricultural University, CHINA

## Abstract

Globally, crustaceans represent one of the most taxonomically diverse and economically important invertebrate group. Notwithstanding, the diversity within this group is poorly known because most crustaceans are often associated with varied habits, forms, sizes and habitats; making species identification by conventional methods extremely challenging. In addition, progress towards understanding the diversity within this group especially in southern Africa has been severely hampered by the declining number of trained taxonomists, the presence of invasive alien species, over exploitation, etc. However, the advent of molecular techniques such as “*DNA barcoding and Metabarcoding*” can accelerate species identification and the discovery of new species. To contribute to the growing body of knowledge on crustacean diversity, we collected data from five southern African countries and used a DNA barcoding approach to build the first DNA barcode reference library for southern African crustaceans. We tested the reliability of this DNA barcode reference library to facilitate species identification using two approaches. We recovered high efficacy of specimen identification/discrimination; supported by both barcode gap and tree-base species identification methods. In addition, we identified alien invasive species and specimens with *‘no ID”* in our DNA barcode reference library. The later; highlighting specimens requiring (i) further investigation and/or (ii) the potential presence of cryptic diversity or (iii) misidentifications. This unique data set although with some sampling gaps presents many opportunities for exploring the effect and extent of invasive alien species, the role of the pet trade as a pathway for crustacean species introduction into novel environments, sea food authentication, phylogenetic relationships within the larger crustacean groupings and the discovery of new species.

## Introduction

Biodiversity loss has emerged as a global concern over the last decades, partly due to human activities [1]. As a result, many nations are taking stringent measures to mitigate this loss, especially in the face of global change. However, current measures have been limited due in part to the unknown extent of global biodiversity, with earlier studies showing that only an approximately 15–18% of all living organisms have been formally described to species level [2–3]. On the other hand, a more recent study using new ecological rules for how biodiversity relates to abundance suggests that there are likely to be at least 1 to 6 billion species on earth see [4] and references therein. Therefore, much progress has been made over the years by taxonomists to discover and describe new species globally, although progress towards any meaningful outcome has been hampered by the declining number of trained taxonomists, and the availability of cryptic species amongst others [5–6]. In the face of declining numbers of trained taxonomists, alternative methods that provide rapid and accurate identification of living organisms have been proposed. Specifically, [7] proposed the use of a short and standardize portion of the genome otherwise known as “DNA Barcodes” for the identification of living organisms. Over a decade and a half since DNA barcoding techniques for specimen identification was established, this method and its associated applications have become increasingly popular in biodiversity science see review by [8] and references therein. For example, DNA barcoding techniques have been widely used to (i) evaluate commercial food product labelling accuracy [9], (ii) identify threatened animal product sold for traditional medicine [10] and particularly wildlife crime [11] etc. Of all these applications, the most prominent has been to document and discover biodiversity within various animal groups including insects, molluscs, fish, mammals etc. [12–14]. However, studies employing the utility of DNA barcodes for species discrimination within crustaceans are still limited but see [15–20]. More so, even with the advent of DNA metabarcoding; a method for assessing biodiversity from environmental samples through bulk DNA extraction, amplification and high throughput sequencing of multiple taxa [21], Carcinologists have not fully taken advantage of this new technique to document and discover new species but see [22]. Yet, crustaceans represent one of the most taxonomically diverse, ecological and economical important invertebrate group [9, 16–17, 20]. For example, crabs, crayfish, lobsters, prawns and shrimps are some of the most expensive and sought-after foods globally. Given their importance, many crustaceans remain under increasing threat from human activities; with the introduction of new species into novel environments increasingly being recognised as a major threat. Such introductions have caused some crustacean species to become established and hence invasive, causing significant negative ecological and economic impacts [23–28]. For example, applying a Generic Impact Scoring System (GISS), a recent study identified the Chinese mitten crab, *Eriocheir sinensis* (Milne-Edwards, 1853) as the most impacting invasive species in Europe based on six environmental and socio-economic categories [29] but see also [30–31] for specific impact of *Balanus glandula* (Darwin, 1854) in South Africa]. With the increasing threats and their global importance, our knowledge within this group globally is still very limited due in part to the fact that most crustaceans are associated with varied habits, forms, sizes and habitats ranging from marine, brackish, to freshwater environments. As a result, crustaceans exhibit a remarkable morphological diversity, involving many potentially cryptic species thus making species identification a challenge. Although previous studies from elsewhere in the world on crustaceans have shown the utility of DNA barcoding techniques for species discrimination in both marine and freshwater systems [15–20, 32], in southern Africa, our knowledge of the crustacean fauna remains limited but see [9]. Therefore, in this study, we aim to build the first DNA barcode reference library of crustacean taxa found in southern Africa (i.e. Angola, Mozambique, Namibia, South Africa, and Swaziland) to facilitate species identification. This reference DNA barcoding library in addition to facilitating species identification will hold enormous potential for the seafood industry, invasive species management, fishing industry etc. in southern Africa and beyond.

## Materials and methods

### Field collections

**Fig 1:**
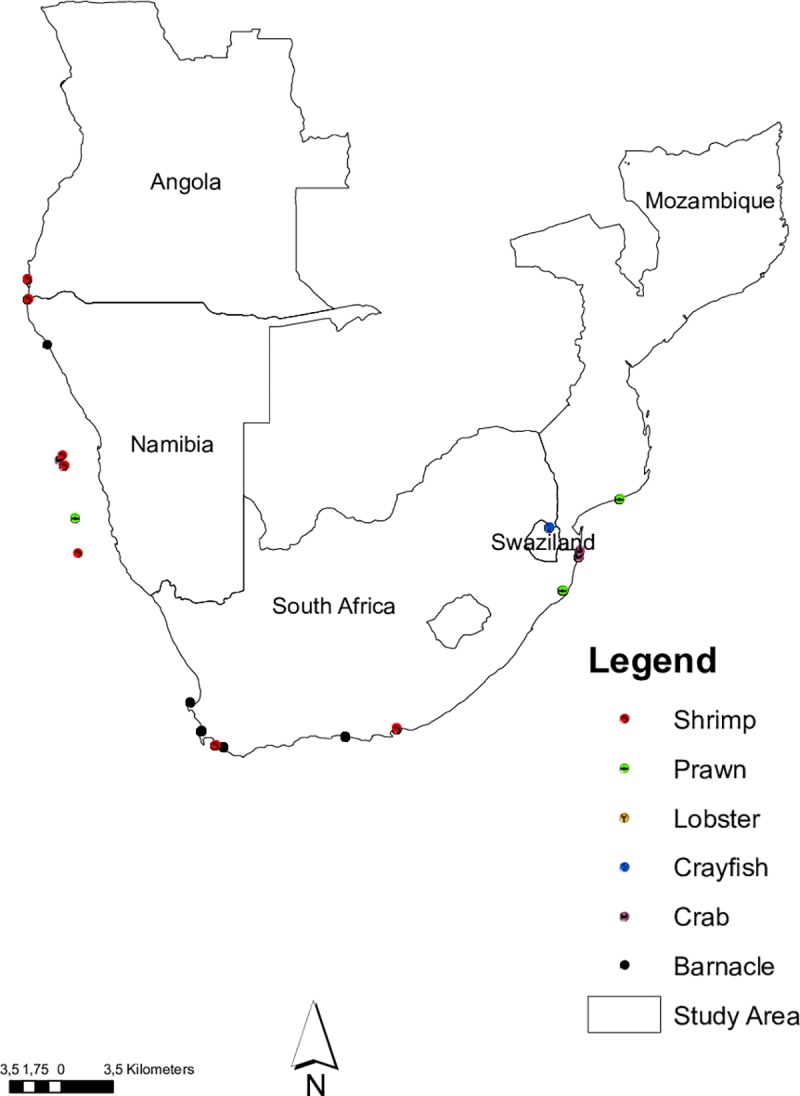
Southern African countries and collection efforts of crustaceans included in our study.

Samples of crustaceans were collected between April 2010 –February 2012 along the southern African coastline and in deep water using trawler boats. Additional samples were collected from freshwater systems (e.g. the Cunenne River mouth). Individual specimens were collected under permit number MAM 18 066202 from the Department of Agriculture, Forestry and Fisheries, South Africa and our studies did not involve endangered or protected species. Specimens were collected from five southern African countries including; Angola, Mozambique, Namibia, Robben Island (South Africa) and Swaziland. Additional samples were collected on a humpback whale; washed off the western coast near Britannia Bay (South Africa; **Fig 1**). Individual specimens were collected under permit number MAM 18 066202 from the Department of Agriculture, Forestry and Fisheries, South Africa.

Morphological species identification was facilitated following expert knowledge, published field guides [25, 33]. All collection details have been logged in public repositories. Specifically, collection details including GPS coordinates, altitude and photographs were deposited in Barcode of Life Data Systems (BOLD; www.boldsystems.org), under the project code HVDBC- Southern African Crustacea.

### DNA extraction, amplification and sequencing

DNA extraction, polymerase chain reactions (PCR) and sequencing of the *COI* gene region (i.e. animal DNA barcode) were done at the Canadian Centre for DNA Barcoding (CCDB) and the African Centre for DNA Barcoding (ACDB) using the primer combination; C_LepFolF and C_LepFolR [34] and following standard protocol as outlined in [35]. For all samples that were successfully amplified, GenBank accession numbers, BOLD process identification numbers, authorities and voucher information are available online in BOLD under the project code HVDBC- Southern African Crustacea.

### Data analyses

Sequence alignment was done using Multiple Sequence Comparison by Log-Expectation MUSCLE version. 3.8.31; [36] and subsequently manually adjusted where necessary. We included in the DNA matrix additional *COI* sequences; including an outgroup retrieved from GenBank database (S1 Table) to test the reliability of our reference library to discriminate amongst species.

### Phylogeny reconstruction

Using the aligned sequence data, we reconstructed the first southern African regional phylogeny for crustaceans. The tree was reconstructed using maximum parsimony (MP) as implemented in PAUP* version. 4.0 [37]. Tree analysis was done by running heuristic searches with 1 000 random sequence additions but keeping only 10 trees per replicate to reduce time spent on branch swapping in each replicate. Tree bisection-reconnection was done with all character transformations treated as equally likely i.e. Fitch parsimony; [38]. MP searches and bootstrap resampling [39] were done to assess node support. Bootstrap support values ranged from: >95% = high support, 70–95% = moderate support and <70% = poor support [39–40]. We used a sea spider, *Nymphon charcoti* (Bouvier 1911) (GenBank ID number: FJ969364), as outgroup to establish monophyly of the species.

### Species identification

We used a combination of approaches to ensure accurate identification of all crustacean species collected. First, we confirmed the identity of the species using morphological characters as per expert opinion and illustrated field guides e.g. [25, 33]. Second, specimen authenticity were verified by BLAST algorithm on GenBank. Last, we used a tree-base identification method to determine the grouping of each species with its close relative(s) on the phylogenetic tree reconstructed [41]. Following this approach, DNA sequences are simulated on the phylogenetic trees and when the query’s conspecific sequences were included in the reference alignment, the rate of positive identification is related to the degree to which different species were genetically differentiated.

### Barcode gap analysis

Using the southern African crustacean phylogeny reconstructed, we evaluated if there exist any significant difference between the mean intra- specific and the mean inter-specific genetic distance based on the Kimura 2-parameter model (K2P) [42–43] for more details on techniques. Lastly, we ran a sequence based simulation analysis as implemented in the R Library SPIDER [44] to test the reliability of the southern African crustacean sequence library to correctly identify species using two criteria; Best Close Match (BCM) and All Species Barcode (ASB). The BCM criterion assigns correct identifications to the closest match regardless of the distance, whereas the ASB criterion simulates the BOLD ID engine by applying a threshold and querying all the sequences within BOLD. First, a species is correctly identified when all the matching sequences below the threshold are conspecific and results are reported as correct when consistent with prior morphological identifications, otherwise the result is incorrect. Second, using the ASB criterion, a query may be reported as ambiguous if sequences divergences of different species are below the threshold (ASB) or sequences from different species are the closest match below threshold (BCM). Last, a query may result in no ID if no match is found below the defined threshold see also [17]. For both BCM and ASB, we used the standard thresholds for BOLD ID engine i.e. 1% K2P; [45]. Additionally, a second threshold was used that minimizes the cumulative identification errors; as implemented in the function ‘threshVal’ in SPIDER. In this approach we identified the sum of false positive (no conspecific matches within threshold of query) and false negative (sequences from multiple species within threshold).

## Results

The DNA barcoding library for southern African crustaceans consist of 174 specimens including seven specimens downloaded from GenBank (S1 Table). We also included a sea spider (*Nymphon charcoti* (Bouvier 1911)) as outgroup. The specimens were distributed as follows: Angola (n = 5) Mozambique (n = 49), Namibia (n = 24), South Africa (n = 84), Swaziland (n = 5) and GenBank (n = 8). Additionally, we identified five invasive species; the whale and rabbit-ear barnacles; *Coronula diadema* (Linnaeus 1767) and *Conchoderma auritum* (Linnaeus 1767), Pacific barnacle; *Balanus glandula* (Darwin 1854), African river prawn; *Macrobrachium vollenhovenii* (Herklots 1857), and redclaw crayfish; *Cherax quadricarinatus* (von Martens 1868). These specimens were spread across 21 families, 29 genera and 33 unique species (**Table 1**).

**Table 1:** Number of specimens representing families, genera, and species included in our reference library.

Native Crustaceans	Families	Genera	Specimens (Unique species)
	16	24	149 (28)
Alien invasive Crustaceans	5	5	25 (5)

**Fig 2:**
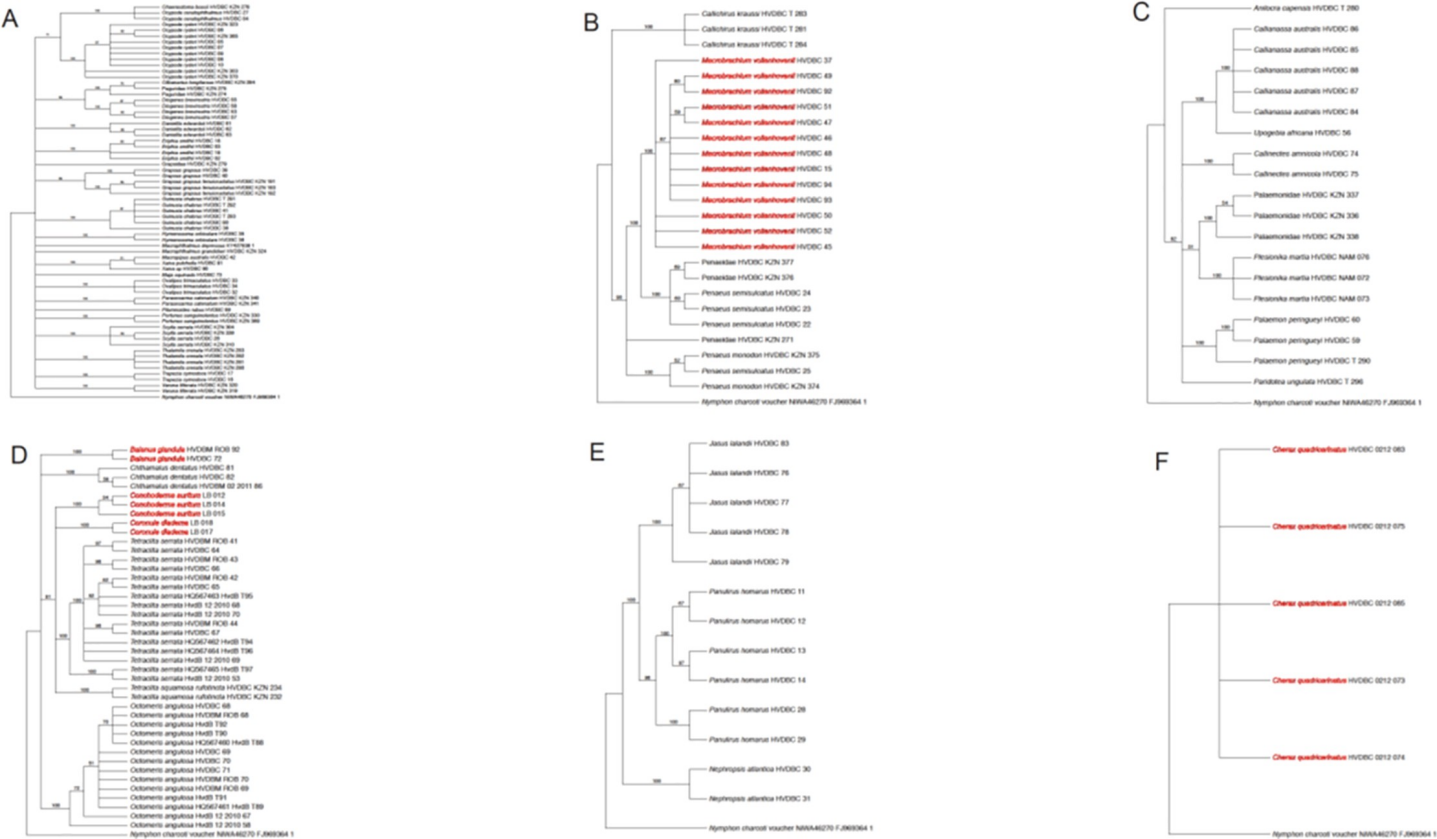
Regional phylogeny of southern African crustaceans (A) Regional phylogeny of crabs (B) Regional phylogeny of prawns (C) Regional phylogeny of shrimps (D) Regional phylogeny of barnacles (E) Regional phylogeny of lobsters (F) Regional phylogeny of crayfish. Species in red indicates the distribution of invasive species on the phylogenetic tree.

The southern Africa regional phylogeny for crustaceans representing 174 species was well supported with moderate to high bootstrap values (see **Fig 2A–2E**). Additionally, we recovered high percentage identification success for southern African crustaceans using both field guides and BLAST algorithm (see **S1 Table**).

### Barcoding gap analysis

**Fig 3:**
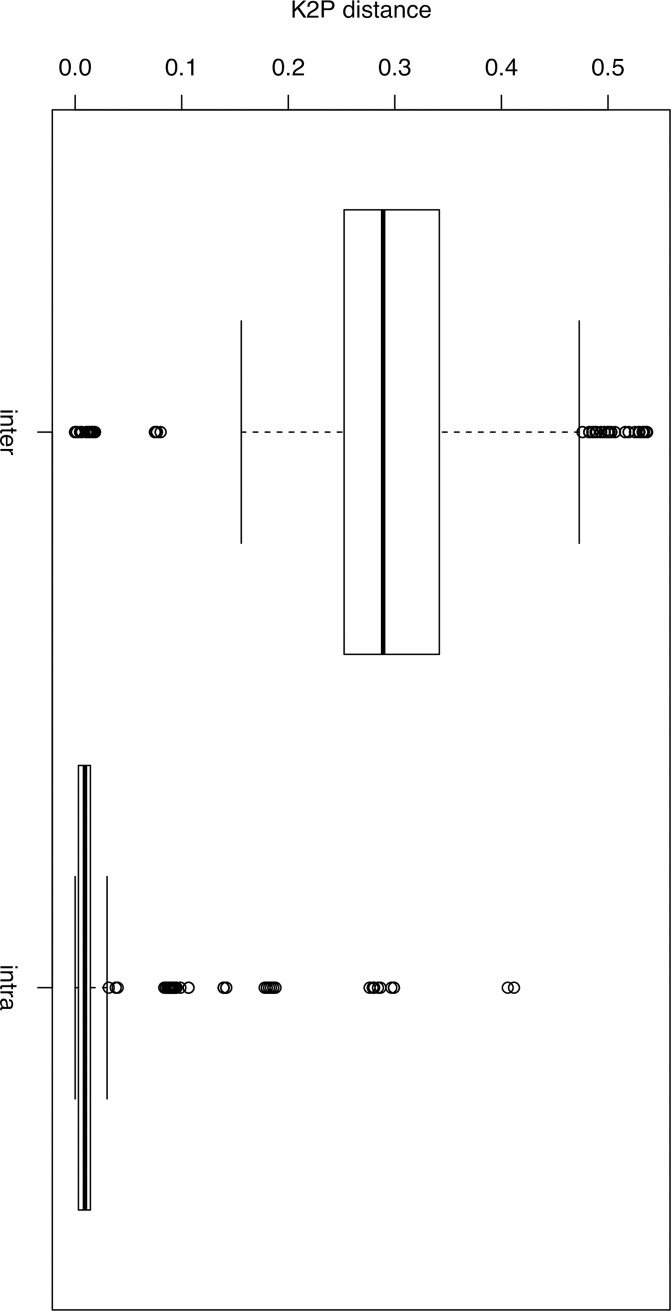
Significantly different genetic distances between and within crustacean species. Box plots represent intraspecific and interspecific genetic distances calculated using Kimura 2-parameter (K2P) model for all species and error bars are interquartile ranges.

Our barcode gap analyses of all individuals belonging to 33 unique species and 21 crustacean families (see **Table 1**) reveals statistically significant differences in K2P values between the mean intraspecific and interspecific comparisons (Mann–Whitney U-test: U = 1106, P < 0.001; **Fig 3**). From the SPIDER analysis and using the BCM criterion, we obtained 143 correct and 5 incorrect identifications (i.e. correct and incorrect identifications indicate positive and negative outcome for this test), 6 ambiguous identifications (i.e. an ambiguous identification refers to the presence of both correct and incorrect identifications or more than one equally close matched species with different identification including the correct one) and 20 no ID (i.e. if query sequence matches were found below the proposed threshold). Using the ASB criterion, we obtained 139 correct and 4 incorrect identifications, 11 ambiguous identifications and 20 no ID. In all analyses, 27 identifications were associated to singletons (i.e. without any conspecific sequences to match). After removing these singletons, incorrect identifications were reduced to two using both criteria.

## Discussion

Crustaceans form one of the most biologically diverse, species rich, nutrient recyclers and economically important arthropod species on Earth. [46] estimated >17,600 living and extinct species of crustaceans, comprising of barnacles, crabs, lobsters, prawns, shrimps etc. but some studies indicate that the actual species diversity within this group of organisms is still several times higher than is presently accepted [47–49]. For example, a more recent article estimates that there exist between 50,000–67,000 known crustaceans globally but scientists estimate the total number of crustaceans to be ten-fold greater than this [50]. This high diversity within crustaceans makes species identification extremely challenging. Although a few morphological characters (i.e. tail length, gill type and number of chelae) have been used in previous studies as key diagnostic features to identify and classify crustaceans [51–53], progress has been slow due to the declining number of trained taxonomists [6]. Nonetheless, the use of modern techniques such as DNA barcoding and metabarcoding can accelerate species identification and discovery [20–21, 35]. In this study, we present the first large scale DNA barcoding library published for southern African crustacean species. From this DNA barcode library, many potential cryptic and alien invasive species were identified, including the whale and rabbit-ear barnacles; *Coronula diadema* (Linnaeus 1767) and *Conchoderma auritum* (Linnaeus 1767) respectively collected from a dead worldwide occurring humpback whale; *Megaptera novaeangliae* (Borowski 1781), washed off the western coast near Britannia Bay (South Africa), Pacific barnacle; *Balanus glandula* (Darwin 1854), African river prawn; *Macrobrachium vollenhovenii* (Herklots 1857), and the Australian redclaw crayfish; *Cherax quadricarinatus* (von Martens 1868) see also [26, 28, 30]. The proliferation of alien invasive crustaceans across water bodies in Africa in general and southern Africa has also been confirmed by some earlier exploratory studies using both taxonomic and DNA barcoding techniques [25–28, 30]. The mere presence of these species and many more undetected species calls for renewed efforts to better understand their biology in these non-native ranges through research; prioritising alien invasive distribution extents, habitat preferences, impacts on native species and local aquatic ecosystems, and management and control. By so doing, rapid response alien mitigation strategies will be developed, which will go long way to minimise the negative impacts of these species on the rich native species diversity. Such strategies will help alien species managers to better prioritise their control and where possible their eradication. Although sampling efforts were concentrated in five southern African countries (see **Fig 1**), we believe the diversity of alien and native species is much higher than current knowledge from the region allows. Exploring the regional phylogeny of southern African crustaceans revealed patterns similar to other studies [54] and references therein. Many previous studies have attempted to resolve the phylogeny of crustaceans, but several challenges exist with many controversies surrounding the internal relationships amongst major taxonomic groups [53–62]. Results from the studies above, including ours (**Fig 2**), indicates that the controversies in resolving the phylogeny of crustaceans remains and the hope to use a single gene to address this challenge is not yet satisfied. This southern African crustacean reference library represents a first step towards using DNA barcoding techniques for the identification of crustacean species in this region. Using the different approaches to identify species (field guide, expert opinion and BLAST), we recovered high efficacy (mean bootstrap support value = 97%) of specimen identification, which was supported by simulations using SPIDER analysis; based on BM and ASB criteria. Also, our reference library reveals the presence of barcoding gap, with the mean interspecific genetic distance being significantly higher than the mean intraspecific genetic distances (P<0.001; **Fig 3**). Furthermore, the mere presence of specimen with *no ID* in our sequence reference library for southern African crustaceans using both criteria mentioned above, highlights specimens requiring (i) further investigation and/or (ii) the potential presence of cryptic diversity or (iii) misidentifications. However, this result should be interpreted with care and not to be confused with *“*species delimitation” [63]. Notwithstanding, some concepts applied here overlap with those used for species delimitation, but results from our simulation analysis exclusively tested the performance of a molecular dataset for identifying represented species.

### Implication for conservation and way forward

Crustaceans consist of one of the most diverse groups of invertebrates with species of high economic values (e.g. crabs, lobsters, and shrimps) and forms the basis of widespread harvesting around the world. With their broad distribution, crustaceans are found in almost all aquatic environments and many species like some crabs have successfully inhabited terrestrial environments. Due to this diversity, species identification has proven challenging for many individuals as very few morphological characters can clearly distinguish between them [52]. This is further compounded by the increasing decline in trained taxonomists around the world and especially in southern Africa [6]. As a result, alternative identification methods have been proposed using molecular sequencing techniques. Specifically, DNA barcoding has been used for over a decade and a half [7–8]. In this study, we use DNA barcoding to generate the first large scale reference library for southern African crustaceans. This unique data set presents many opportunities for exploring the effect and extent of invasive alien species, the role of the pet trade as a pathway for crustacean species introduction into new environment, sea food authentication, phylogenetic relationships within the larger crustacean groupings and the discovery of new species [9, 23–24, 26, 53, 64]. More exploratory studies are encouraged especially from the less known African waters; taking advantage of new developments in the field of biodiversity science (artificial intelligence, remote sensing, DNA metabarcoding etc.) to fill the data and knowledge gaps identified in this study.

## Conclusions

The data set presented here, although with some sampling gaps, represents an important first step towards establishing a complete DNA reference library for southern African crustaceans. Even though there exist some challenges in utilising DNA barcoding techniques, for example its high sensitivity to environmental contaminants, our reference library holds great potential for species identification, invasive species biomonitoring, sea food authentication and taxonomic revision of major groups amongst others. Notwithstanding, with the advancements in DNA barcoding and metabarcoding technologies [21], crustacean surveys in particular and the biomonitoring of alien and native species in general will proceed faster since metabarcoding techniques have been successful at detecting taxa even at low abundance from environmental samples, whereas there were unnoticed by conventional taxonomic and DNA barcoding methods. This is reflected in our study area, where we found some alien invasive species including *Balanus glandula* (an invasive pacific barnacle arguably introduced through ballast water from ships coming from the Pacific North America).

## Supporting information

S1 Table Supplementary table highlighting all sequence IDs generated by this study and downloaded from GenBank and percentage specimen identification. (XLSX)
